# Effects of high fructose corn syrup on intestinal microbiota structure and obesity in mice

**DOI:** 10.1038/s41538-022-00133-7

**Published:** 2022-03-02

**Authors:** Xiaorong Wang, Liying Zhu, Xiaoqiong Li, Xin Wang, Ruirong Hao, Jinjun Li

**Affiliations:** 1 College of Animal Science, Shanxi Agricultural University, Taigu, 030801 P. R. China; 2 Institute of Food Sciences, Zhejiang Academy of Agricultural Sciences, Hangzhou, 310021 P. R. China; 3 State Key Laboratory for Managing Biotic and Chemical Threats to the Quality and Safety of Agro-products, Zhejiang Academy of Agricultural Sciences, Hangzhou, 310021 P. R. China

**Keywords:** Risk factors, Microbial ecology

## Abstract

High fructose corn syrup (HFCS)-associated health problems have raised concerns. We investigated the effects of HFCS-containing drinking water on body fat, intestinal microbiota structure of mice, and the relationships between them. HFCS drinking water significantly increased body fat content and altered the intestinal microbiome. The *Christensenellaceae R-7 group* negatively correlated with body weight, perirenal fat, epididymal fat, and liver fat percentage.

## Introduction

High fructose corn syrup (HFCS) has gradually become a prominent form of sugar in the human diet in recent years due to its advantages over sucrose in texture, taste, preparation methods, and cost^1^. As HFCS is widely used as a sweetener in various beverages and food products, its excessive consumption has become a major health issue. Intake of beverages and food products containing HFCS is associated with changes in systemic and tissue-specific metabolic status, which result in profound effects on the body, such as causing obesity^2^, insulin resistance-related and obesity-induced non-alcoholic fatty liver disease (NAFLD)^3^, cardiovascular diseases^4^, type 2 diabetes mellitus (T2DM)^5^, reproductive system diseases^6^, and even cancer^7^.

The numerous microbes present in the intestinal tracts of humans and mammals regulate many physiological functions, including proteins, fats, and carbohydrates metabolism, the production of vitamins and minerals essential for the body, the maintenance of intestinal integrity and intestinal barrier function, regulation of immune functions, and protection against pathogens. Disturbances to the microbiota in a healthy intestinal tract might result in the onset of various chronic diseases. Intestinal microbiota participates extensively in lipid metabolism and the onset and development of obesity and is associated with metabolic diseases, such as NAFLD, cardiovascular diseases, obesity, T2DM, and metabolic syndrome^8^.

Current research on the effects of HFCS on humans or animals has mainly focused on metabolic conditions, such as NAFLD and diabetes mellitus, with few studies investigating its effects on gut microbiota changes and composition. Therefore, we employed 16 S rDNA sequencing analysis to determine the effects of HFCS ingestion on intestinal microbiota diversity and composition in mice. By analyzing the correlations between body weight, perirenal fat weight, epididymal fat weight or liver fat percentage, and the colonic microbiota structure, we also deduced the possible mechanisms by which changes in intestinal microbiota structure induce obesity signs.

## Results

### Effects of HFCS on intestinal microbiota structure and obesity in mice

**Fig. 1:**
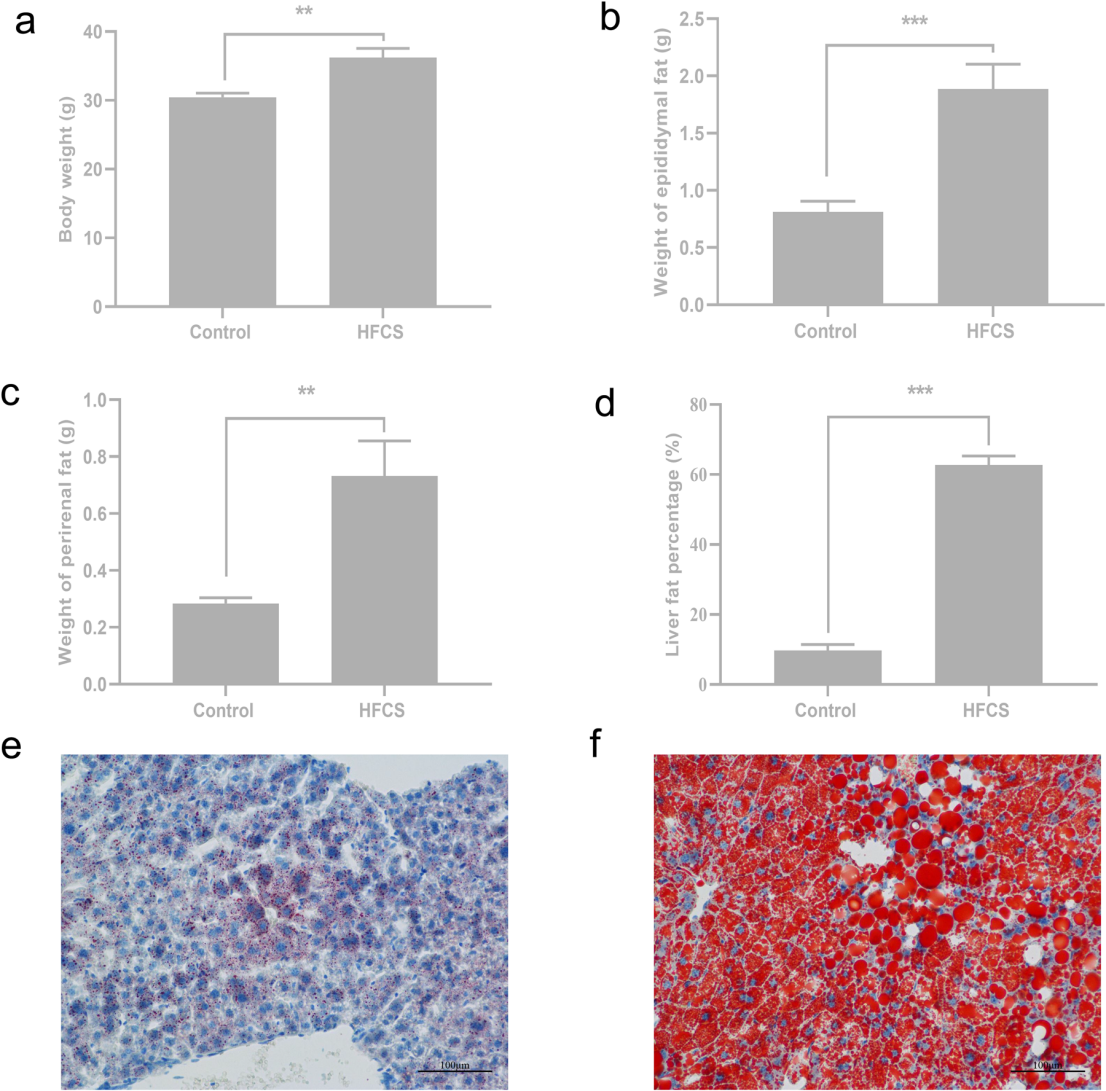
Effects of high fructose corn syrup (HFCS) on the obesity phenotype in mice. **a** Body weight; **b** weight of epididymal fat; **c** weight of perirenal fat; **d** liver fat percentage; **e**, **f** Oil red O-stained liver tissues. All date are expressed as the mean ± SEM. *n* = 8. *p < 0.05, **p < 0.01, ***p < 0.001 (two-sided *t*-test).

**Fig. 2:**
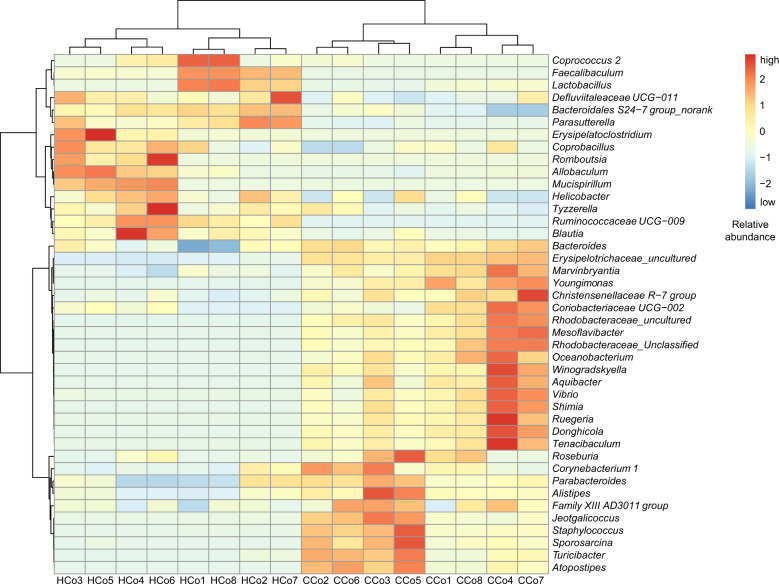
Hierarchically clustered heat map for the significantly different bacterial genera in colon of mice. CCo represents the colon of the control group, HCo represents the colon of the HFCS group. Software and algorithm: R language vegan package, vegdist and hclust for distance calculation and cluster analysis.

In this study, the long-term intake of drinking water containing 30% HFCS caused significant increases in body weight, perirenal fat, epididymal fat (*P* = 0.001, *P* = 0.0009, *P* = 0.007, respectively), and liver fat percentage in mice (*P* < 0.0001), as shown by a considerable increase in the proportion of area occupied by positively stained lipid droplets (Fig. 1). Further, high-throughput sequencing revealed the occurrence of significant changes in the colonic microbiota. The community richness of colonic microbiota in the HFCS group was significantly decreased compared with that of the control group (Supplementary Table 1). Beta-diversity analysis revealed the presence of significantly different clusters between the two groups (Supplementary Fig. 1). At the phylum level, the taxonomic distribution of the colonic microbial communities in both groups displayed that Firmicutes, Bacteroidetes, Proteobacteria, Actinobacteria, Tenericutes, and Deferribacteres were (Supplementary Fig. 2a) the dominant bacteria, which accounted for more than 99.29% of the colonic microbiota. Changes in colonic microbiota composition at the genus level were also observed (Supplementary Fig. 2b). In both groups, the top 10 genera with the highest relative abundances were *Erysipelotrichaceae_unculture*d, *Bacteroidales S24-7 group_norank*, *Allobaculum*, *Faecalibaculum*, *Staphylococcus*, *Lactobacillus*, *Bacteroides*, *Lachnospiraceae_uncultured*, *Turicibacter*, and *Lachnospiraceae NK4A136*. The bacteria with higher relative abundance and significant difference between the two groups were screened and plotted on a heat map (Fig. 2). This indicates that the intake of HFCS-containing drinking water resulted in considerable changes in the colonic microbiota structure of mice.

### Correlations between microbial genera and obesity indices

From the 42 genera with significant differences in relative abundance between the two groups, the obesity-associated genera *Christensenellaceae R-7 group* and *Tyzzerella*, as well as the pathogenic bacteria *Erysipelatoclostridium* and *Helicobacter*, were selected to analyze their correlations with phenotypic variables of body weight, perirenal fat, epididymal fat, and liver fat percentage (Supplementary Fig. 3). We found that *Tyzzerella*, *Erysipelatoclostridium*, and *Helicobacter* were positively correlated with body weight, perirenal fat, epididymal fat, and liver fat percentage, whereas *Christensenellaceae R-7 group* was strongly negatively correlated. The genus *Tyzzerella* of the family Lachnospiraceae showed a significant increase in abundance after intervention with HFCS-containing drinking water. At the genus level, the relative abundances of conditional pathogenic bacteria, such as *Erysipelatoclostridium* and *Helicobacter*, were significantly increased in the HFCS group compared with those in the control group.

## Discussion

### HFCS may induce obesity by affecting *Christensenellaceae R-7 group* in intestinal flora

The increase in body weight and visceral fat was consistent with previous studies^9,10^. The accumulation of visceral fat leads to a higher tendency of insulin resistance, disorders in sugar utilization, and increased lipolysis, which are key factors for the onset of metabolic syndrome. Therefore, the measurement and analysis of body weight, visceral fat content, and peripheral fat content are of great importance^11^. Wostmann et al. found that the increase in body weight of mice in a germ-free environment was slower than that of normal mice, which demonstrates the beneficial effects of microbial communities on fat deposition^12^. Other researchers have also directly demonstrated the relationships between microbiota and metabolic disorders, such as obesity^13^. In mice on a high-fat diet and obese individuals reportedly have higher abundances of Firmicutes and Bacteroidetes in the body, forming an obesity-type intestinal microbiota^14^, but other studies have reported that a high-fat diet reduces the ratio of Firmicutes to Bacteroidetes and the abundances of intestinal microbes^15^. The results of the present study might be attributed to the fact that mice of the HFCS group were only provided HFCS-containing drinking water and basal feed without the addition of high-fat feed. Firmicutes are capable of utilizing carbohydrates in food for the synthesis of butyrate, which serves as an energy source and maintains epithelial cell morphology and normal function in the colon^16^. Pediatric researchers at the University of Alberta in Canada found that Lachnospiraceae species feed on undigested carbohydrate fibers in the human body, thereby contributing additional calories through carbohydrate degradation and resulting in obesity^13^. Christensenellaceae, a member of the phylum Firmicutes, is abundant in the colons of humans and animals and accounts for 0.01% of the fecal microbial communities^17^. Christensenellaceae are also closely associated with host health as their relative abundance is associated with the onset of colorectal cancer and inflammatory bowel disease^18–21^. Further, the relative abundance of this family in the intestines is associated with body mass index, a direct indicator of obesity, in the populations of many countries, which includes adult men and women of different age groups^17^. The existence of a negative correlation between Christensenellaceae and the amount of visceral fat has also been reported^22^. Metabolic disorders are usually related to dietary patterns, with research evidence indicating that *Christensenellaceae R-7 group* is decreased in populations on a refined sugar diet^23^. Such evidence is consistent with the results of the present study, which showed that long-term high-dose HFCS consumption led to a decrease in *Christensenellaceae R-7 group*, thereby inducing obesity or even the inflammatory response in mice. These results are consistent with those reported by Beaumont et al^17,22^. and demonstrate that the relative abundance of *Christensenellaceae R-7 group* is negatively correlated with body weight and visceral fat content. Therefore, it can be deduced that HFCS intake induces obesity in mice by regulating the relative abundance of the genus *Christensenellaceae R-7 group*.

The results of this study provide theoretical support for the rational use of sweeteners, suggesting that HFCS may induce obesity by affecting *Christensenellaceae R-7 group* in intestinal flora. However, the specific mechanism is still unclear, which will be further studied in the follow-up study.

## Methods

Sixteen male specific pathogen-free grade C57BL/6J mice aged 3 weeks and weighing 18–22 g each were purchased from Shanghai SLAC Laboratory Animal Co., Ltd. (Shanghai, China) [License No. SCXK(Lu)2017-0005]. The study was approved by the Zhejiang Provincial Ethics Committee for Laboratory Animals (Ethical Approval No. 78865576).

The mice were randomly divided into the control group and HFCS group (8 each) after one week of acclimatization. All animals were reared in a clean environment at the Laboratory Animal Center of Zhejiang Academy of Agricultural Sciences (Animal Experimentation License No. 286868667) under the following conditions: rearing temperature, 21 ± 2 °C; relative humidity, 50–80%; 12-h light/dark cycle, and ad libitum access to food and water. Co60-irradiated normal feed (18.6% crude protein (mass %), 4.8% crude fat, 61% carbohydrate) was purchased from Jiangsu Xietong Pharmaceutical Bio-engineering Co., Ltd. (Jiangsu, China). HFCS (F55, i.e., 55% fructose and 45% glucose) was purchased from COFCO (Chengdu) Grains and Oils Industries Co., Ltd. (Sichuan, China).

The control and HFCS groups were given pure water and 30% (w/v) HFCS^24^, respectively, in addition to the normal diet. After 16 weeks of rearing, the mice were anesthetized and dissected. The colon of each mouse was removed, and the colonic contents were collected into a sterile centrifuge tube and stored in a − 80 °C freezer prior to intestinal microbiota analysis. The liver segments were fixed in 4% paraformaldehyde, and then frozen slices were made. The slices were stained with Oil Red solution (Servicebio) and examined under microscope. Genomic DNA was extracted from the colonic contents using the QIAamp DNA Stool Mini Kit (QIAGEN, Valencia, CA, USA) and confirmed by 1% agarose gel electrophoresis. Based on the specified sequencing regions, specific barcoded primers were synthesized for the amplification of the V3 + V4 regions of the 16S rDNA gene. The agarose gel was sliced to recover the amplification products, which were quantified using the QuantiFluor™ system and sequenced using the HiSeq 2500 PE250 platform at Mingke Biotechnology (Hangzhou) Co., Ltd after purification. Raw sequencing data were subjected to raw data filtering, tag assembly, tag filtering, and the removal of chimeric tags to eliminate low-quality data and obtain effective tags for subsequent analysis. Operational classification unit (OTU) analysis was performed on non-repeated sequences according to 97% similarity. Species matching was performed for all representative sequences of OTU using RPD databases. Principal component analysis was used to analyze beta diversity among samples. Pie charts are used to show taxa at the phylum and genus levels.

All experimental data were statistically analyzed using SPSS 26.0 and plotted using GraphPad. Differences were compared using the t-test and results were expressed as the mean and standard error of the mean; * denotes *P* < 0.05, ** denotes *P* < 0.01, and *** denotes *P* < 0.001.

## Supplementary information

Supplementary_information

## Data Availability

The authors declare that the data supporting the findings of this study are presented within the manuscript. The sequences obtained in the study were deposited in the NCBI Sequence Read Archive under accession number PRJNA 744020. Additional data sources are also available from the corresponding author upon reasonable request.
