# Supplementary material for: Effects of high fructose corn syrup on intestinal microbiota structure and obesity in mice
Source: NPJ Sci Food. 2022 Mar 2;6:17. doi: 10.1038/s41538-022-00133-7 (PMC8891263; doi:10.1038/s41538-022-00133-7)
Supplement: Supplementary file 1 — Supplementary_information [file 41538_2022_133_MOESM1_ESM.pdf]

SUPPLEMENTARY INFORMATION

Effects of high fructose corn syrup on intestinal microbiota structure and obesity in mice

Xiaorong Wang [1,2], Zhu Liying[2], Xiaoqiong Li[2], Xin Wang[2, 3], Ruirong Hao [1]*,Jinjun Li [2]*

[1] College of Animal Science,Shanxi Agricultural University,Taigu 030801, P. R. China.
[2] Institute of Food Sciences, Zhejiang Academy of Agricultural Sciences, Hangzhou 310021, P. R. China.
[3] State Key Laboratory for Managing Biotic and Chemical Threats to the Quality and Safety of Agro-products, Zhejiang Academy of Agricultural Sciences, Hangzhou, 310021, P. R. China;
* Correspondence: hrr823229@126.com (R. H.); lijinjun@zaas.ac.cn (J. L.)

Supplementary Table 1

Supplementary Figure 1-3

Primer sequences

Supplementary Table 1. Sequencing results of the control and high fructose corn syrup (HFCS) groups

Item	OTUs	Chao1 index	Coverage	Shannon index	Simpson index
Control	348.3	412.75	0.998	3.04	0.15
HFCS	280.5	325.75	0.999	2.98	0.14
SEM	10.61	12.86	0.0001	0.05	0.011
P-value	<0.001	<0.001	<0.001	0.585	0.634

Supplementary Table 1 shows that the coverage of both groups was >0.99, indicating that species diversity was well-represented in the samples. Results of alpha diversity analysis showed that the number of operational taxonomic unit (OTUs) and Chao index of the control group were significantly higher than those of the HFCS group ($P<0.05$); in other words, community richness was higher in the control group than in the HFCS group. Differences in the Shannon and Simpson indices between the two groups were not statistically significant.

Supplementary Figure 1

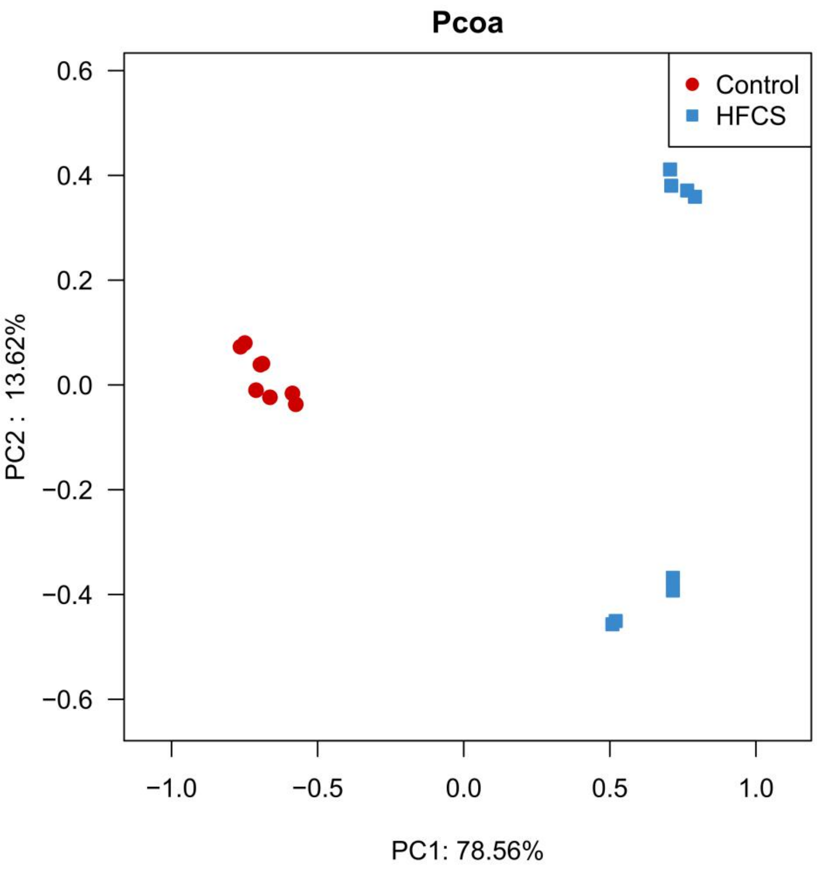

Supplementary Figure 1 Principal coordinates analysis (PCoA) of the colonic microbiota structure of mice. HFCS, high fructose corn syrup.

Supplementary Figure 2

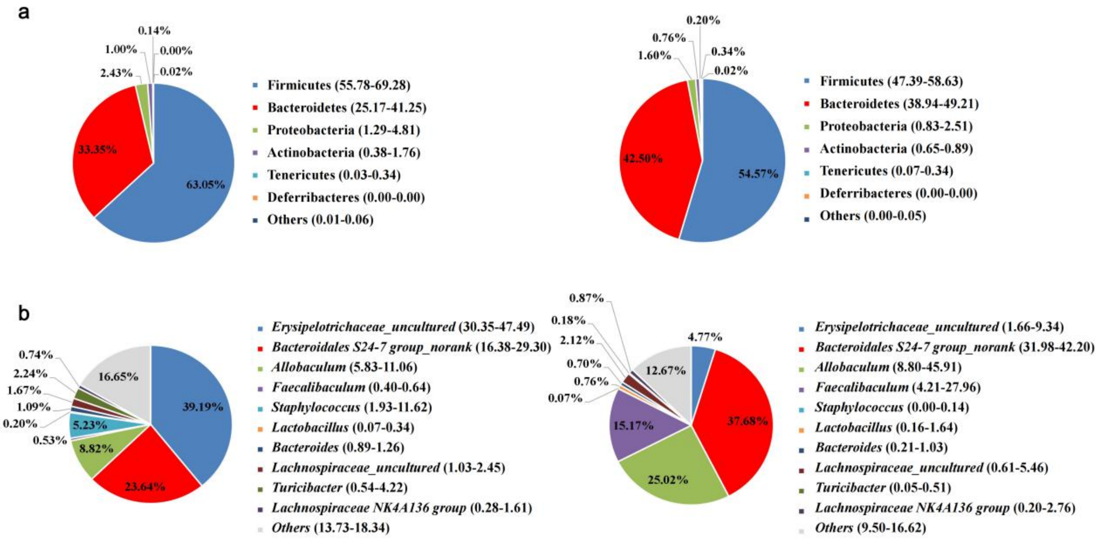

Supplementary Figure 2 Effects of high fructose corn syrup (HFCS) on the colonic microbiota of mice. Pie charts of the distribution of colonic microbial communities in the control and HFCS groups at the phylum (a) and genus (b) levels. Only the top six phyla and top 10 genera with the highest relative abundances are shown.

Supplementary Figure 3

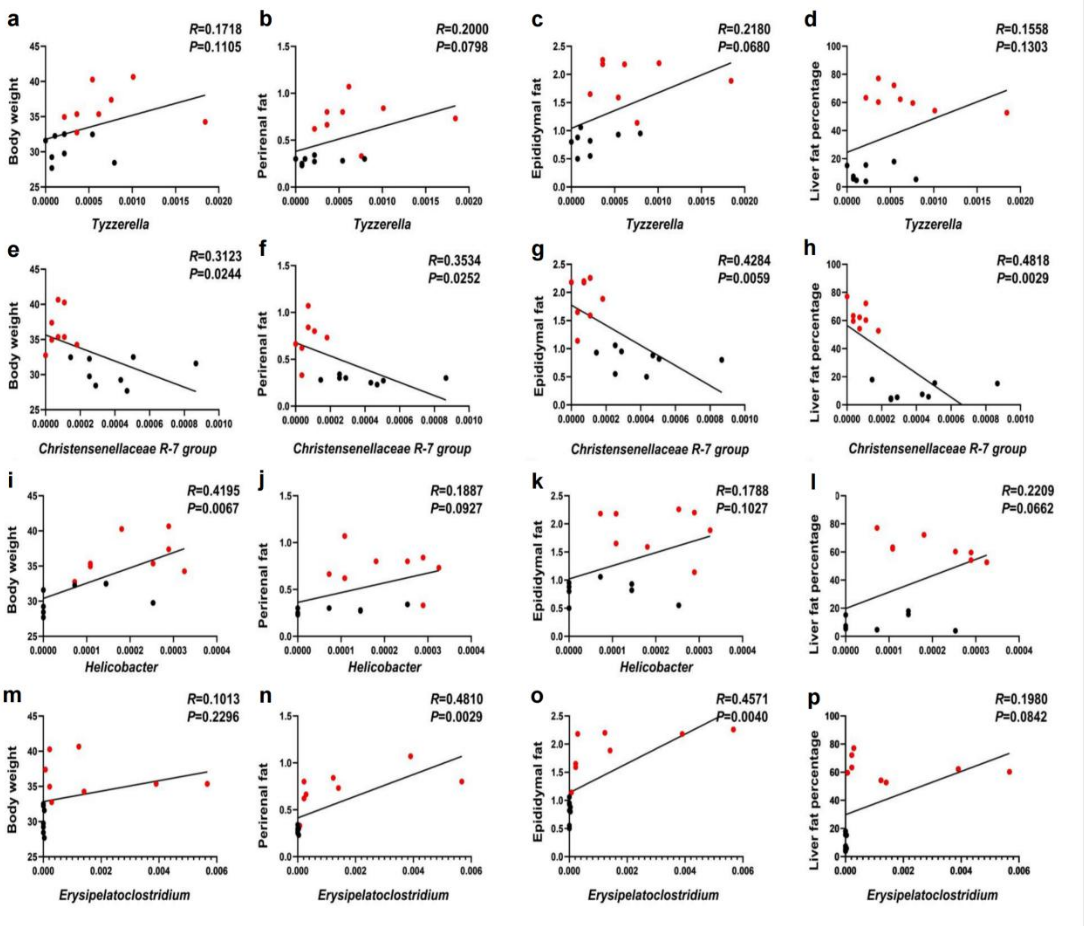

Supplementary Figure 3 Correlation analysis of microbial genera and obesity indices. a–d, e–h, i–l, m–p indicate the correlations between *Tyzzerella*, *Christensenellaceae R-7 group*, *Erysipelatoclostridium*, or *Helicobacter* and body weight, perirenal fat, epididymal fat, and liver fat percentage.

The primer sequences were as follows:
515F: 5′-GTGCCAGCMGCCGCGGTAA-3′;
907R: 5′-CCGTCAATTCMTTTRAGTTT-3′.
